# Single and combined association between brominated flame retardants and cardiovascular disease: a large-scale cross-sectional study

**DOI:** 10.3389/fpubh.2024.1357052

**Published:** 2024-03-26

**Authors:** Wenhao Yin, Rui Xu, Jiyu Zou, Yaqin Wang, Yan Zhang

**Affiliations:** ^1^Department of Cardiovascular Medicine, Liaoning University of Traditional Chinese Medicine, Shenyang, China; ^2^Department of Respiratory Medicine, Liaoning University of Traditional Chinese Medicine, Shenyang, China; ^3^The Affiliated Hospital of Liaoning University of Traditional Chinese Medicine, Shenyang, China

**Keywords:** brominated flame retardants, cardiovascular diseases, joint exposure, NHANES, cross-sectional study, PBB153

## Abstract

**Introduction:**

The single and combined association between brominated flame retardants (BFRs) and cardiovascular diseases (CVD) has remained unelucidated. This research aimed at exploring the associations between mixture of BFRs and CVD.

**Methods:**

This research encompassed adult participants from the National Health and Nutrition Examination Survey in 2005–2016. The weighted quantile sum (WQS) model and quantile g-computation (QGC) model were applied to examine the combined effects of BFRs mixture on CVD.

**Results:**

In this research, overall 7,032 individuals were included. In comparison with the lowest quartile, the highest quartile of PBB153 showed a positive association with CVD, with odds ratio (OR) values and 95% confidence intervals (CI) of 19.2 (10.9, 34.0). Furthermore, the acquired data indicated that PBB153 (OR: 1.23; 95% CI: 1.02, 1.49), PBB99 (OR: 1.29; 95% CI: 1.06, 1.58), and PBB154 (OR: 1.29; 95% CI: 1.02, 1.63) were linked to congestive heart failure. PBB153 was also related to coronary heart disease (OR: 1.29; 95% CI: 1.06, 1.56). Additionally, a positive correlation between the BFRs mixture and CVD (positive model: OR: 1.23; 95% CI: 1.03, 1.47) was observed in the weighted quantile sum (WQS) model and the quantile g-computation (QGC) model.

**Discussion:**

Therefore, exposure to BFRs has been observed to heighten the risk of cardiovascular disease in US adults, particularly in the case of PBB153. Further investigation is warranted through a large-scale cohort study to validate and strengthen these findings.

## 1 Introduction

Cardiovascular disease (CVD) encompasses a range of heart and blood vessel diseases, including heart failure, angina pectoris, coronary heart disease, heart attack, and stroke (1). It is the foremost cause of fatality globally, with total deaths attributed to CVD increasing by 21.1% between 2007 and 2017 (2). Multiple risk factors have been identified in epidemiological studies as contributors to CVD and related mortality. These encompassed obesity/overweight, inadequate physical activity, poor diet, smoking status, uncontrolled blood pressure, and diabetes among adults (3). In addition to recognized risk factors, recent studies have highlighted the role of certain chemicals and toxins in increasing the risk of CVD (4–6). For instance, research has observed that isopentanaldehyde can increase the risk of CVD by raising triglyceride levels and white blood cell count (4). Similarly, a modest association has been found between levels of polycyclic aromatic hydrocarbons and CVD in US adults (6). Nevertheless, it is important to highlight the existing research gap concerning the association between BRFs and the risk of CVD. To comprehensively grasp the potential effect of these chemicals on cardiovascular health, additional exploration and investigation are imperative.

BFRs found widespread applications in various products such as plastics, textiles, furniture, and electronics (7). Additionally, they have also been found in other food or human medium such as seafood, poultry, breast milk, serum, urine, and hair (8–10). The toxicity, persistence, accumulation, and biomagnification of BFRs have raised concerns (11). Categorization of BFRs as per their incorporations into polymers results in divisions such as reactive agents, brominated monomers, and additive agents. Among these, the reactive BFRs like polybrominated diphenyl ethers (PBDEs) and 2,2′,4,4′,5,5′-hexabromobiphenyl (PBB153) are associated with adverse effects on the environment and human health (12). Although efforts are being made to phase out the use of BFRs, they are still present in durable consumer products, food (13), indoor dust (14, 15), and occupational environments (16). As reported, a significant body of research has shown that BFRs are linked to detrimental health outcomes, including thyroid disorders, neurobehavioral and developmental disorders, and reproductive health issues (17–19). Therefore, the impact of BFRs on human health cannot be ignored in the future.

The impact of exposure to BFRs on the onset or progression of CVD has been the subject of multiple studies. However, the resulting data have only provided weak or inconclusive evidence regarding this association. Only a single study was successful in reporting that PBB153 was correlated with cardiovascular mortality. However, the strength of these findings was constrained by the type of BFRs and statistical models used (20). Previous studies typically investigated the association between BFR exposure and CVD by utilizing a single pollutant model, thereby allowing the investigation of each BFR individually. However, since individuals are simultaneously exposed to multiple chemicals, the combined effects of such exposures may differ from the sum of the effects of a single pollutant (21). Additionally, the single-pollutant model is vulnerable to confounding factors resulting from exposure to other pollutants and lacks the capability to assess potential interactions between these exposures. Therefore, it is crucial to address the challenges associated with investigating the health implications of exposure to a mixture of chemicals. Specifically, a critical aspect is the examination of the potential impact resulting from simultaneous exposure to a mixture of BFRs on CVD. This research can yield valuable insights for public health interventions and provide a more accurate understanding of the complex interactions between environmental exposures and health outcomes.

To elucidate this process further, a comprehensive analysis was executed via advanced statistical techniques. Herein weighted quantile sum regression (WQSR) and quantile g-computation (QGC) were utilized to investigate the association between maternal exposure to eight different types of BFRs and CVD based on US population-based data.

## 2 Method

### 2.1 Study population

**Figure 1:**
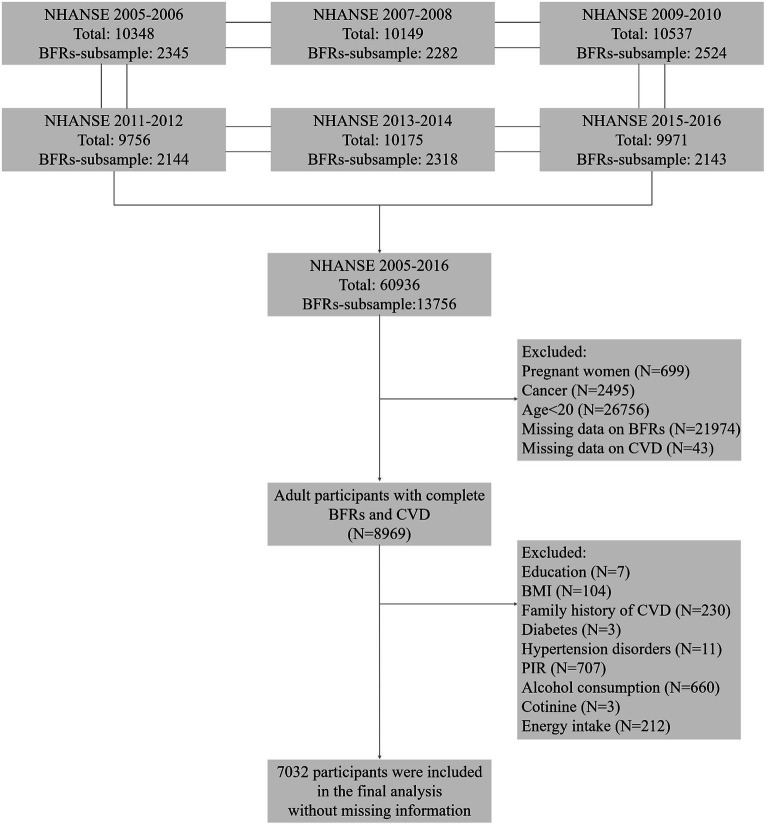
Flow chart.

The National Health and Nutrition Examination Survey (NHANES) is a comprehensive study that collected data through personal structured, health examinations, and laboratory analyzes in the US. The survey recruited a representative sample of the U.S. population with a stratified, multistage probability design (22). In this research, data from NHANES 2005–2016 were utilized, which provided information on concentrations of BFRs in serum. To facilitate the analysis of these chemicals, a weighted pooled-sample design was employed, which involved combining samples before conducting specific analytical measurements. A total of 60,936 participants were enrolled at first. Pregnant women (*N* = 699), cancer patients (*N* = 2,495), individuals aged below 20 years old (*N* = 26,756), individuals with missing complete data on BFRs (*N* = 21,974), CVD (*N* = 43), and covariates (*N* = 1937) were excluded. Ultimately, 7,032 participants were subjected to the final analysis in Figure 1.

### 2.2 Exposure: BFRs

In the NHANSE dataset, PBB153 and PBDEs were measured in serum. This process involved automated liquid–liquid extraction and subsequent sample clean-up. This study focused on PBB-153 and seven PBDEs whose detection rate exceeded 75% (23). The seven PBDEs included PBDE28, PBDE47, PBDE85, PBDE99, PBDE100, PBDE153, and PBDE154. For values that fell below the limit of detection (LOD), imputation was performed using the square root of two.

### 2.3 Outcomes: CVD

Cardiovascular disease was assessed through individual interviews using self-reported physician diagnoses and standardized medical status questionnaires. The participating individuals were asked to answer a series of questions, including “Have you ever been informed by a doctor or other health professional that you have coronary heart disease (CHD), congestive heart failure (CHF), heart attack, angina pectoris, or stroke?” Participants with affirmative answers, i.e., “yes” to any of these answers were categorized as CVD patients (6). To simplify the analysis, the outcome variable was converted into a dichotomous variable, with participants being classified as either having CVD or not having CVD.

### 2.4 Covariates

Participants in this study were assessed for various covariates encompassing age, sex, race/ethnicity, family income, educational level, energy intake, physical activity, natural logarithm transformed concentrations of cotinine, BMI, hypertension, diabetes mellitus, and family history of CVD. These covariates were included to control for potential confounding factors and to adjust for their impacts on the association between BFRs and CVD. Age was categorized as either 50 years old or younger or older than 50. Gender was categorized as men or women, and race was divided into Mexican American, Other Hispanic, Non-Hispanic White, Non-Hispanic Black, and Other Races. Family income, as measured by the income-to-poverty ratio (PIR), was classified as either below 1.0 (a low family income) or ≥ 1.0 (a high family income). Educational level was categorized as less than 9th grade, 9th–11th grade, high school graduate/GED or equivalent, some college or AA degree, and college graduate or above. The categories utilized for assessing energy intake were inadequate, adequate, or excessive, and were based on predefined cutoffs. Males with energy intake below 2000 kcal/day or females with energy intake below 1,600 kcal/day were defined as having inadequate energy intake. Conversely, males with energy intake above 3,000 kcal/day or females with energy intake above 2,400 kcal/day were defined as having excessive energy intake. Physical activity levels were classified as higher intensity, lower intensity, or no physical activity. We extract data based on five items that are consistently unchanged from NHANES 2005–2016, including Vigorous work activity (Yes vs. No), Moderate work activity (Yes vs. No), Walk or bicycle (Yes vs. No), Vigorous recreational activities (Yes vs. No), and Moderate recreational activities (Yes vs. No). If the answer is Yes, the value is 1 point; if the answer is No, the value is 0 points. We calculate the total score of the five items, if the total score is 0, it is no physical activity, if the total score is 1 or 2, it is low intensity physical activity, and if the total score is more than 3, it is high intensity physical activity. The concentrations of cotinine, a biomarker of tobacco smoke exposure, were ln-transformed for analysis. Body mass index (BMI) was determined as per the given formula; weight (kg) divided by height (meters squared). The individuals were categorized as normal weight (BMI < 24.9), overweight (24.9 ≤ BMI < 29.9), and obesity (BMI ≥ 29.9). Hypertension and diabetes mellitus were determined based on self-reported diagnosis, with participants categorized as having or not having these conditions. Lastly, participants were categorized as having a family history of CVD or not based on self-reported information.

### 2.5 Statistical analysis

Survey-weighted multiple logistic regression analysis was performed to explore the relationship between BFRs and the prevalence of CVD. Continuous variables were expressed as mean ± SD, while categorical variables were presented as weighted percentages. Three models were established: Model 1 (crude model), Model 2 (adjusted for age, gender, race, and education), and Model 3 (adjusted for variables in Model 2 along with physical activity, ln-transformed cotinine, consumption of alcohol, CVD family history, hypertension disorders, diabetes, energy intake, BMI, and PIR). Subgroup analysis was executed via multivariate logistic regression, with stratification based on factors such as age, gender, BMI, physical activity, consumption of alcohol, energy intake, CVD family history, diabetes, and hypertension disorders. To examine and evaluate the heterogeneity of the association across various subgroups, an interaction term was incorporated. This evaluation was executed via the log-likelihood ratio test model.

Herein, a comprehensive analysis of the association between multiple BFRs and the outcome variable was performed through two statistical methods: Weighted Quantile Sum (WQS) regression and quantile g-computation analysis (QGC). WQS regression is a multivariable approach that considers the combined effects of all predictor variables on the outcome. It determines the contribution of each variable to the overall effect by calculating their relative weights. The parameters were estimated using a weighted index, and the training data were divided, with 40% allocated for model development and the remaining 60% utilized for validation. The robustness of the acquired data was ensured by applying 1,000 bootstrap samples. The QGC analysis is a technique that allows the assessment of the synergistic effects arising from exposure to multiple chemicals. It allows the researcher to make unbiased inferences about mixture effects with appropriate confidence interval coverage. This is possible due to the integration of the simplicity of WQS regression with the flexibility of g-computation. In this analysis, specific weights were assigned to the BFRs, which represented their proportionate contribution to the overall mixture exposure. Each exposure was assigned a range of 0–1, and the positive and negative weights were summed to 1.

The statistical analyzes were performed using R 4.1.2 (Core Team, Vienna, Austria). A *p*-value less than 0.05 denoted statistical significance, indicating a strong association between the BFRs and the outcome variables.

## 3 Results

### 3.1 Characteristics

This study included a total of 7,032 individuals, and the overall weighted prevalence of CVD was found to be 6.3%. Table 1 presents the weighted baseline parameters of the population under study. The acquired data indicated that individuals with CVD had a higher likelihood of being advanced age (82.4% vs. 36.4%), non-Hispanic and other race (91.6% vs. 85.7%), having an education level below high school (53.6% vs. 38.0%), having lower family PIR (17.9% vs. 13.5%), being overweight or obese (50.4% vs. 36.4%), consuming inadequate energy (55.3% vs. 34.0%), having lower alcohol consumption (29.7% vs. 22.0%), engaging in no physical activity (50.4% vs. 26.9%), have a family history of CVD (26.0% vs. 11.9%), and experience hypertension (73.8% vs. 26.2%) or diabetes (32.1% vs. 6.8%). However, the data did not exhibit any significant variation between individuals with CVD in terms of gender and ln-transformed cotinine (a biomarker of smoking exposure) in urine. Moreover, individuals with CVD were observed to have a greater likelihood of exposure to elevated levels of several BFRs, including PBDE27, PBDE48, PBDE85, PBDE99, PBDE100, PBDE153, and PBB153. The median values, interquartile range (IQR) values, and detection ratios of these BFRs in the enrolled participants are shown in Supplementary Table S1 for different cycles. The acquired data also observed a decreasing trend in the concentrations of BFRs over the increasing years. Supplementary Table S2 presents the overall detection ratio of the BFRs in the assessed individuals. Additionally, Supplementary Figure S1 displays the correlations across the various substances, with the strongest relationship observed between PBDE85 and PBDE99 (*r* = 0.93).

**Table 1:** Weighted population characteristics by cardiovascular disease, National Health and Nutrition Examination Survey (NHANES) 2005–2016.

Characteristics	All participants (7,032)		*P*-value
	Non-CVD (6,440)	CVD (592)	
Age, %			<0.001
<50	3,869 (63.6)	79 (17.6)	
≥50	2,571 (36.4)	513 (82.4)	
Gender, %			0.363
Male	3,114 (49.0)	329 (52.0)	
Female	3,326 (51.0)	263 (48.0)	
Race, %			<0.001
Mexican American	1,112 (8.7)	70 (4.9)	
Other Hispanic	642 (5.6)	41 (3.5)	
Non-Hispanic White	2,706 (67.9)	287 (72.1)	
Non-Hispanic Black	1,358 (11.5)	164 (15.4)	
Other race	622 (6.3)	30 (4.1)	
Education, %			<0.001
Less than 9th grade	602 (4.9)	85 (8.6)	
9–11th grade	912 (10.0)	108 (17.4)	
High school graduate/GED or equivalent	1,453 (23.1)	157 (27.6)	
Some college or AA degree	1912 (31.3)	159 (28.3)	
College graduate or above	1,561 (30.7)	83 (18.1)	
PIR, %			0.023
<1.0	1,316 (13.5)	146 (17.9)	
≥1.0	5,124 (86.5)	446 (82.1)	
BMI, (kg.m^−2^), %			<0.001
<24.9	1842 (30.3)	108 (18.0)	
24.9–< 29.9	2,162 (33.3)	193 (31.6)	
≥29.9	2,436 (36.4)	291 (50.4)	
Physical activity, %			<0.001
Higher intensity	1,490 (17.1)	64 (18.0)	
Lower intensity	3,558 (56.0)	297 (31.6)	
No	1,392 (26.9)	231 (50.4)	
Energy intake, (kcal/day), %			<0.001
Inadequate	2,438 (34.0)	365 (55.3)	
Adequate	2,771 (45.4)	184 (36.3)	
Excessive	1,231 (20.6)	43 (8.4)	
Alcohol consumption, %			0.001
Yes	4,669 (78.0)	399 (70.3)	
No	1771 (22.0)	193 (29.7)	
Family history of CVD, %			<0.001
Yes	722 (11.9)	140 (26.0)	
No	5,718 (88.1)	452 (74.0)	
Diabetes, %			<0.001
Yes	609 (6.8)	212 (32.1)	
No	5,831 (93.2)	380 (67.9)	
Hypertension disorders, %			<0.001
Yes	1883 (26.2)	456 (73.8)	
No	4,557 (73.8)	136 (26.2)	
PBDE28, pg./g^a^	6.8 (4.6, 10.2)	7.9 (5.6, 13.3)	<0.001
PBDE47, pg./g^a^	114.2 (78.9, 188.3)	148.3 (91.5, 241.3)	<0.001
PBDE85, pg./g^a^	2.2 (1.4, 4.0)	2.9 (1.7, 5.0)	<0.001
PBDE99, pg./g^a^	21.8 (14.1, 37.2)	29.6 (15.4, 49.5)	<0.001
PBDE100, pg./g^a^	24.1 (15.3, 39.0)	27.4 (17.2, 48.9)	<0.001
PBDE153, pg./g^a^	57.5 (36.1, 91.2)	52.1 (33.4, 94.5)	0.001
PBDE154, pg./g^a^	2.0 (1.4, 3.7)	2.6 (1.7, 4.6)	0.174
PBB153, pg./g^a^	14.6 (7.3, 27.2)	24.13(17.0, 40.6)	<0.001
Ln-transformed cotinine, pg./g^a^	−3.2 (−4.5, 2.5)	−3.3 (−4.5, −0.4)	0.127

a Median (IQR).

CVD, Cardiovascular disease; PIR, Ratio of family income to poverty; BMI, Body mass index.

### 3.2 Single model

Table 2 presents the associations between ln-transformed concentrations of BFRs and CVD. In the crude model, individuals in the highest quartile of PBB153, PBDE28, PBDE47, PBDE85, PBDE99, PBDE100, and PBDE154 had a remarkably elevated risk of CVD compared to those in the lowest quartile. The odds ratios (OR) and 95% confidence intervals (CI) for these associations were as follows: PBB153, 19.2 (10.9, 34.0); PBDE28, 2.83 (1.96, 4.08); PBDE47, 1.97 (1.39, 2.80); PBDE85, 1.77 (1.24, 2.54); PBDE99, 1.79 (1.27, 2.54); PBDE100, 1.88 (1.32, 2.66); and PBDE154, 1.79 (1.27, 2.53) (*p* < 0.01). Conversely, PBDE153 was negatively associated with CVD, with an OR of 0.70 (0.52, 0.95) for the third quartile compared to the first quartile. After adjusting for all covariates, the positive association between PBB153 and CVD remained significant. Individuals in the fourth quartile of PBB153 had a 4.84-fold (2.49, 9.40) increased risk of CVD in comparison to their first-quartile counterparts. Similarly, individuals in the third and second quartiles of PBB153 also had significantly higher risks of CVD, with ORs of 5.11 (2.74, 9.56) and 3.72 (1.99, 6.95), respectively (*p* < 0.001). Additionally, we assessed the association the lipid-adjusted concentrations of BFRs and CVD in Supplementary Table S3.

**Table 2:** Associations of with ln-transformed serum BFRs with CVD in all participants.

	Model 1^a^	Model 2^b^	Model 3^c^
PBB153			
Q1	Ref (1.0)	Ref (1.0)	Ref (1.0)
Q2	8.17 (4.66, 14.3)^***^	4.01 (2.13, 7.54)^***^	3.72 (1.99, 6.95)^***^
Q3	17.6 (10.0, 31.0)^***^	5.87 (3.08, 11.2)^***^	5.11 (2.74, 9.56)^***^
Q4	19.2 (10.9, 34.0)^***^	5.86 (2.97, 11.6)^***^	4.84 (2.49, 9.40)^***^
PBDE28			
Q1	Ref (1.0)	Ref (1.0)	Ref (1.0)
Q2	1.65 (1.12, 2.44)^*^	1.17 (0.76, 1.81)	1.18 (0.72, 1.93)
Q3	1.50 (1.05, 2.14)^*^	0.98 (0.66, 1.46)	0.99 (0.63, 1.54)
Q4	2.83 (1.96, 4.08)^***^	1.56 (1.04, 2.35)^*^	1.53 (0.97, 2.42)
PBDE47			
Q1	Ref (1.0)	Ref (1.0)	Ref (1.0)
Q2	1.07 (0.77, 1.48)	0.91 (0.65, 1.29)	0.82 (0.57, 1.19)
Q3	1.57 (1.11, 2.22)^*^	1.25 (0.86, 1.80)	1.18 (0.78, 1.78)
Q4	1.97 (1.39, 2.80)^***^	1.37 (0.95, 1.99)	1.28 (0.85, 1.93)
PBDE85			
Q1	Ref (1.0)	Ref (1.0)	Ref (1.0)
Q2	0.88 (0.62, 1.26)	0.91 (0.63, 1.31)	0.89 (0.60, 1.30)
Q3	1.37 (0.98, 1.91)	1.20 (0.84, 1.71)	1.21 (0.83, 1.75)
Q4	1.77 (1.24, 2.54)^**^	1.30 (0.90, 1.87)	1.20 (0.79, 1.80)
PBDE99			
Q1	Ref (1.0)	Ref (1.0)	Ref (1.0)
Q2	0.81 (0.57, 1.16)	0.79 (0.55, 1.12)	0.73 (0.49, 1.08)
Q3	1.61 (1.15, 2.23)^**^	1.32 (0.94, 1.87)	1.30 (0.90, 1.90)
Q4	1.79 (1.27, 2.54)^**^	1.31 (0.91, 1.89)	1.21 (0.80, 1.82)
PBDE100			
Q1	Ref (1.0)	Ref (1.0)	Ref (1.0)
Q2	1.33 (0.95, 1.86)	1.13 (0.79, 1.62)	1.06 (0.72, 1.57)
Q3	1.17 (0.82, 1.69)	1.02 (0.70, 1.47)	0.98 (0.66, 1.45)
Q4	1.88 (1.32, 2.66)^***^	1.32 (0.90, 1.92)	1.22 (0.80, 1.87)
PBDE153			
Q1	Ref (1.0)	Ref (1.0)	Ref (1.0)
Q2	0.84 (0.58, 1.21)	0.94 (0.64, 1.38)	0.89 (0.60, 1.33)
Q3	0.70 (0.52, 0.95)^*^	0.75 (0.53, 1.05)	0.76 (0.53, 1.10)
Q4	0.83 (0.58, 1.19)	0.80 (0.56, 1.16)	0.76 (0.51, 1.12)
PBDE154			
Q1	Ref (1.0)	Ref (1.0)	Ref (1.0)
Q2	0.97 (0.69, 1.35)	0.79 (0.56, 1.11)	0.75 (0.52, 1.08)
Q3	1.38 (0.95, 2.01)	1.00 (0.69, 1.47)	1.02 (0.69, 1.51)
Q4	1.79 (1.27, 2.53)^**^	1.26 (0.88, 1.81)	1.18 (0.77, 1.79)

a Model 1: crude model.

b Model 2: adjusted for age, gender, race, and education.

c Model 3: Model 2 + physical activity + ln-transformed cotinine + alcohol consumption + family history of CVD + diabetes + hypertension disorders + energy intake + BMI + PIR.

^***^ *P* < 0.001; ^**^ *P* < 0.01; ^*^ *P* < 0.05.

Additional analyzes were performed to explore the relationship between BFRs and CVD in different subgroups. Among individuals with congestive heart failure, it was observed that PBB153, PBDE99, and PBDE154 were associated with increased risk using both ln-transformed concentrations of serum BFRs and ln-transformed lipid-adjusted concentrations of serum BFRs in Supplementary Table S4. Specifically, for ln-transformed concentrations, the ORs were observed to be 1.23 (95%CI: 1.02, 1.49) for PBB153, 1.29 (95%CI: 1.06, 1.58) for PBDE99, and 1.29 (95%CI: 1.02, 1.63) for PBDE154. For lipid-adjusted concentrations, the corresponding ORs were 1.25 (95%CI: 1.03, 1.52) for PBB153, 1.26 (95%CI: 1.01, 1.56) for PBDE85, 1.32 (95%CI: 1.08, 1.61) for PBDE99, and 1.34 (95%CI, 1.05, 1.70) for PBDE154.

The associations between PBB153 and CVD were investigated further by taking into account various covariates and using ln-transformed concentrations in Supplementary Table S5. The analysis was executed separately for the different subgroups of the covariates. It was observed that the increased risk of CVD associated with PBB153 was more pronounced in overweight individuals in comparison to those with normal weight (OR: 4.87, 95%CI: 1.74, 13.6 vs. OR: 3.80, 95%CI: 1.02, 14.1; *p* for interaction = 0.03). Additionally, individuals without diabetes mellitus exposed to PBB153 were at a higher risk of developing CVD in comparison to those with diabetes mellitus (OR: 8.71, 95%CI: 4.00, 19.0 vs. OR: 1.19, 95%CI: 0.38, 3.37; *p* for interaction<0.001). Furthermore, when analyzing the associations between PBB153 and CVD using lipid-adjusted concentrations, the data implied that participants with a family history of CVD, when exposed to PBB153, were at a higher risk of developing CVD (OR: 6.64, 95%CI: 2.10, 21.0; *p* for interaction = 0.043). The data acquired imply that the association between PBB153 and CVD may vary depending on factors such as weight status, diabetes mellitus, and family history of CVD. Additionally, we also the associations between lipid adjusted concentrations of PBB153, PBDE28, PBDE153 and CVD stratified by covariates in Supplementary Tables S6–S8 separately.

A non-linear relationship between PBB153 and CVD was observed (*p* for nonlinearity <0.05). Specifically, the data indicated that PBB153 had a pronounced inverted U-shaped relationship with CVD. In contrast, the other seven BFRs showed a linear relationship with CVD that reached a plateau (Supplementary Figure S2).

### 3.3 Combined model

**Figure 2:**
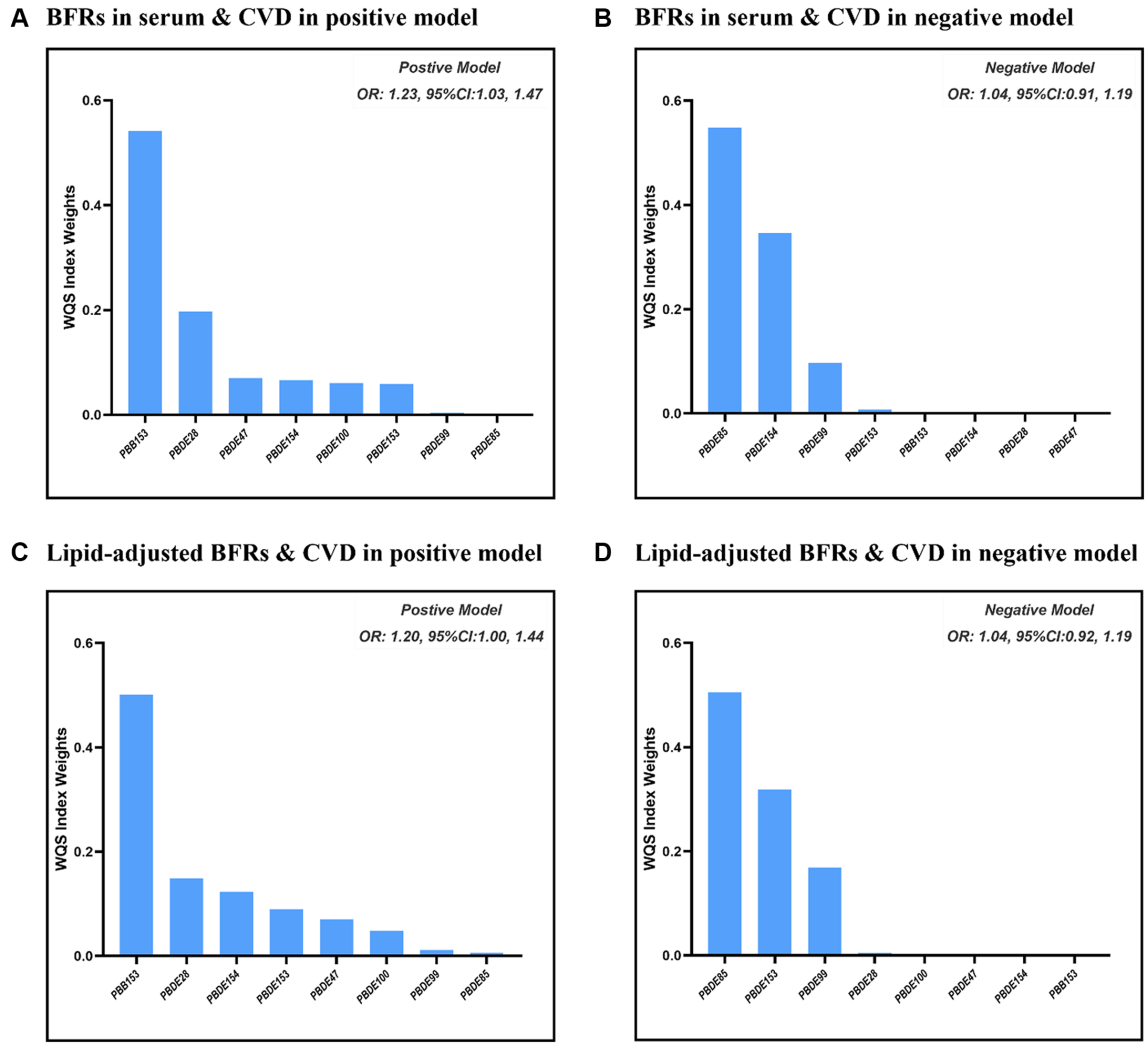
Combined association between BFRs and CVD using WQS model. **(A)** ln-transformed concentration of BFRs in positive model; **(B)** ln-transformed concentration of BFRs in negative model; **(C)** ln-transformed concentration of BFRs in lipid in positive model; **(D)** ln-transformed concentration of BFRs in lipid in negative model.

**Figure 3:**
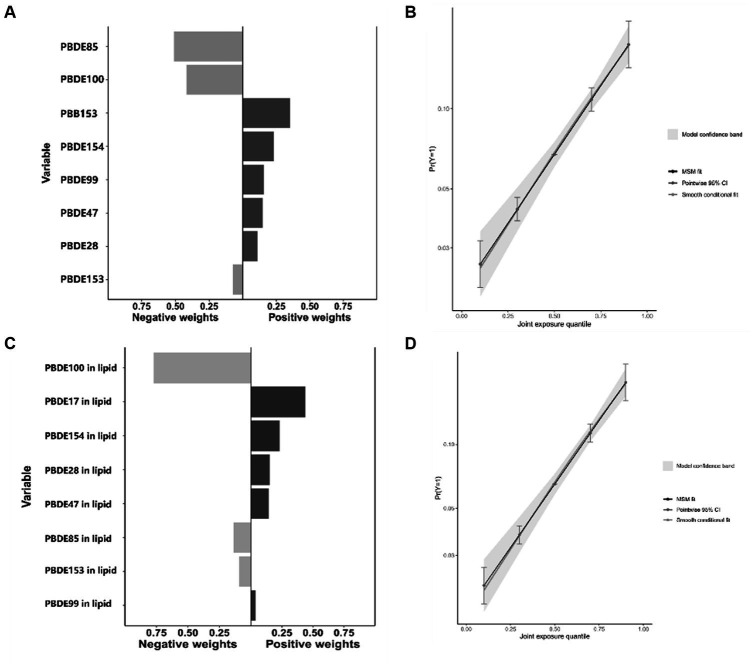
Combined association between BFRs and CVD using QGC model. **(A)** Proportion of ln-transformed concentration of BFRs in QGC model; **(B)** combined effects of ln-transformed concentration of BFRs in QGC model; **(C)** proportion of ln-transformed concentration of BFRs in lipid in QGC model; **(D)** combined effects of ln-transformed concentration of BFRs in lipid in QGC model.

Figure 2 presents the results of the covariate-adjusted associations between serum BFR concentrations and CVD and its components using the WQS regression analysis. When analyzing in the positive direction, the WQS index showed a significant positive association with CVD using ln-transformed BFR concentrations (OR: 1.23; 95% CI: 1.03, 1.47) and lipid-adjusted concentrations (OR: 1.20; 95% CI: 1.00, 1.44). Among the individual BFRs, PBB153, PBDE28, and PBDE47 were found to have a relatively stronger impact on CVD. It was observed that PBDE99 and PBDE85 had the lowest contribution to the overall associations of CVD. However, no significant association was observed when analyzing in the negative direction using ln-transformed concentrations (OR: 1.04; 95% CI: 0.91, 1.19) and lipid-adjusted concentrations (OR: 1.04; 95% CI: 0.92, 1.19). The QGC model yielded similar results, showing the overall mixture effect estimates of BFRs contributions to the outcome (psi1 beta coefficients, 95%CIs, and the *p*-values) for the combined association. Panels B and D showed the results for the positive association between serum BFRs (QGC *β*: 0.2837; 95% CI: 0.1401, 0.4273, *p* < 0.0001) and lipid-adjusted serum BFRs (QGC *β*: 0.098; 95% CI: 0.075–0.120, *p* < 0.0001) concentrations, and CVD in Figure 3. Panels A and C also showed the weights representing the proportion of the positive or negative partial effect for each BFR in the quantile g-computation model for CVD. In serum BFRs, PBB153, PBDE154, PBDE99, PBDE47, and PBDE28 presented positive weights, whereas PBDE85, PBDE100, and PBDE153 had a negative weight. In lipid-adjusted BFRs, PBDE17, PBDE154, PBDE28, PBDE47, and PBDE99 presented positive weights, whereas PBDE100 PBDE85, and PBDE153 had a negative weight.

## 4 Discussion

As per the analysis of population-based representative data, multiple logistic regression, WQSR, and GQC models were utilized to examine the relationships between serum concentrations of BFRs and CVD. The data acquired in this research implied that certain BFRs remained elevated in human serum even after being discontinued from the US market for an extended period of time. Notably, PBB153 emerged as a significant chemical in relation to overall CVD, particularly congestive heart failure and coronary heart disease. Additionally, remarkable positive correlations were observed between BFRs and the risk of CVD and its various components. These correlations exhibited different shapes, encompassing linear and inverted U-shaped patterns with a plateau. Furthermore, these associations were found to differ based on factors such as BMI, diabetes mellitus, and family history of CVD.

Legacy BFRs are still exposed in various ways despite being phased out (7). Compared with other countries, exposure to BFRs tended to be higher in the United States (24). Particularly for PBB153, recent evidence suggests that this compound is absent from the air in regions like Asia, Africa, Latin America, and the Pacific (25). In some contexts, the discovery of low concentrations of PBB153 may seem encouraging, implying its global disappearance. However, it is crucial to recognize that the adverse effects stemming from long-term past exposure are indeed significant and should not be disregarded, as reported in our study. Recent findings indicate that animals may be susceptible to the effects of BFRs on their hearts (26–28). In this research, the acquired data implied that the cumulative impact of BFRs elevated the CVD risk. Of all the components studied, PBB153 emerged as the most influential, elevating the risk of developing CVD in a distinctive U-shaped pattern. Prior research on the impact of BFR exposure on CVD has been relatively scarce. To date, only one study, performed by Kristiann et al., reported that PBB153 was linked to cardiovascular mortality (OR:1.13, 95% CI: 1.00, 1.27, *p* = 0.05). Additionally, they observed that the other BFR components were not remarkably linked to cardiovascular mortality (20). This earlier study marked the first demonstration of a relationship between PBDEs and cardiovascular mortality in the population. However, the association between BFRs and CVD was not stronger than in our study. Earlier studies have pointed out that the concentration of PBB153 tends to increase with a higher initial PBB level, younger age at exposure, higher BMI, lower education, current smoking, and heavy alcohol use (29, 30). In comparison to the research by Kristiann et al., participants in our study exhibited a tendency toward higher exposure levels of PBB153. The study was further expanded by incorporating PBDE28, PBDE85, PBDE99, and BPDE154 into a comprehensive sample from NHANES spanning from 2005 to 2016. These specific compounds were not evaluated in the study by Kristiann et al., as their analysis was limited to data collected up to NHANES 2003–2004. In line with previously observed nonlinear relations, it was found that PBB153 exhibited a distinct inverted U-shape association with CVD. A review assessing receptor-mediated responses in a biological system revealed that initially, these responses may intensify with the dosage increase. However, it was observed that these responses may dimmish with subsequent dose increases. BFRs, due to their status as endocrine disruptors, may exhibit varying dose–response curves under various exposure patterns in epidemiological studies (31). Furthermore, it is important to note that the majority of BFRs seemed to exhibit non-toxic properties. Nevertheless, there remains a lingering concern that these compounds, or potential contaminants in BFR mixtures could interact with cellular components (32). This research utilized both the WQS regression and the QGC models in order to examine whether serum BFR mixtures had any impact on CVD. This approach addresses the limitations of conventional statistical methods, such as the challenge of conducting single comparisons, managing multicollinearity, and dealing with high dimensionality. Thus, our study offers further insight into the association between BFRs and CVD, utilizing robust statistical methods and a larger, more recent dataset.

Epidemiology studies have demonstrated that PBDE was associated with adverse hemodynamic patterns, including higher heart rate, shortened pre-ejection period, and lower total peripheral resistance. These patterns indicate an elevated risk of developing CVD (33). However, in the population studied in this research, PBDEs were not found to be associated with CVD in adults, even after full adjustment for covariates. It is important to note that variations in results may occur due to differences in the exposure stages (children vs. adults) (33), choice of statistical models, and any undetected covariates. In addition, BRFs have been demonstrated to be significantly correlated with numerous cardiovascular risk factors (23, 34–36). For example, Lu et al. (36) reported that BFR mixtures were found to have adverse effects on oxidative stress markers, with PBB153 being linked to elevated levels of bilirubin (*β*: 0.042, 95% CI: 0.022, 0.063) and gamma-glutamyl transferase (*β*: 0.042, 95% CI: 0.022, 0.063). A cross-sectional study observed that PBB153 heightened the risk of Metabolic syndrome significantly (OR: 2.38; 95% CI: 1.73, 3.30) (23). Additionally, Zhang et al. (37) studied the data of 150 Chinese females and found that TG and total PBDEs were positively correlated in a recent cross-sectional study. Firstly, BFRs may exacerbate metabolic syndrome by directly reacting with DNA, proteins, and lipids, disrupting the normal structure of cells and affecting the metabolic pathways and cell fate of endothelial cells and smooth muscle cells (17, 38). And finally, BFRs can also stimulate the body to produce more reactive oxygen species and free radicals, resulting in increased levels of oxidative stress in the body, which may further damage vascular endothelial cells and cause cardiovascular disease (39). Therefore, the possible mediating role of metabolic syndrome and oxidative stress in these associations should be observed further.

In line with our acquired data, previous studies have both substantiated the correlation between BFRs with CVD *in vitro* and *in vivo*. For example, chronic PBB administration resulted in decreased hemoglobin and cardiac output, the voltage amplitude of the ECG, and a shift in the mean electrical axis in chicks (40). Previous studies further reported that exposure to BFRs in serum was related to significant enrichment of genes and metabolomic markers involved in cellular respiration and lipid metabolism (41, 42). Glucose metabolism and lipid metabolism are vital in regulating myocardial energy metabolism (43). Therefore, metabolism reprogramming might be a potential mechanism by which BFRs contribute to CVD. *In vitro*, numerous studies provided compelling evidence that BFRs contribute to the development of diseases by disrupting various processes such as pro-inflammatory cytokines, lipid metabolism, vascular endothelial cell function, and foam cell formation (44).

This research has several strengths. Firstly, multiple chemical exposures were assessed in a large population in the US, which helped to reduce the influence of confounding chemicals. Secondly, different mixture modeling methods were employed to investigate the association between BFRs and CVD. This allowed the exploration of various research questions and enhanced the reliability of the conclusions reached. Thirdly, potentially important factors were rigorously controlled during the assessment. Comprehensive data on demographic and socioeconomic attributes, medical histories, and lifestyle, were acquired, enabling us to adjust for potential covariates.

Nevertheless, it is essential to acknowledge certain limitations in this study. Firstly, as a cross-sectional study, causation cannot be definitively established. The causal direction remains uncertain–whether CVD leads to heightened exposure to BFRs, or if increased BFR exposure triggers CVD. To delve into the temporal relationship between BFR exposure and CVD, further longitudinal investigations are warranted. Secondly, despite our identification of a positive association between the BFR mixture and CVD, the underlying processes responsible for the varying effects of different BFRs cardiovascular diseases, leading to inconsistent positive weights or negative weights, require a more comprehensive examination. Furthermore, this study did not investigate potential interactions between various BFRs. Hence, further research is imperative to comprehend how these chemicals may interact with one another. Thirdly, it is important to note that this research did not incorporate specific novel BFRs developed as replacements for PBDEs. This exclusion was primarily attributed to the absence of relevant data within the NHANES dataset. To offer a more in-depth understanding of the link between BFR exposure and CVD, future studies should consider incorporating these novel BFRs. Finally, while efforts were made to adjust for numerous measured confounding factors, the presence of undetected confounding cannot be completely ruled out, especially in the case of co-exposed pollutants and cardiovascular related diseases (including valvular disease, arrhythmia, cardiomyopathy, pulmonary hypertension and other diseases). Consequently, additional research is warranted to improve the control of potential confounders and assess their impact on the association between BFR exposure and CVD.

## 5 Conclusion

In conclusion, although BFRs have been phased out already, the adverse outcomes caused by BFRs should not be ignored. A significant finding in this research is that the majority of BFRs remain detectable in the general US population. It was observed that as overall BFR exposure increased, so did the risk for CVD and its components. Notably, PBB153 emerged as a significant chemical in relation to CVD, holding significant implications for safeguarding individuals against BFR pollution and its associated cardiovascular risks. Furthermore, it is essential to delve deeper into the potential mechanisms underlying this association.

## Data availability statement

The original contributions presented in the study are included in the article/Supplementary materials, further inquiries can be directed to the corresponding author.

## Ethics statement

The studies involving human participants were reviewed and approved by NCHS Research Ethics Review Board (ERB). Written informed consent for participation was not required for this study in accordance with the national legislation and the institutional requirements.

## Author contributions

WY: Formal analysis, Methodology, Software, Visualization, Writing – original draft. RX: Conceptualization, Data curation, Formal analysis, Validation, Writing – original draft. JZ: Conceptualization, Software, Supervision, Validation, Writing – original draft. YW: Methodology, Supervision, Validation, Writing – review & editing. YZ: Funding acquisition, Resources, Supervision, Writing – review & editing.
