# Supplementary material for: Single and combined association between brominated flame retardants and cardiovascular disease: a large-scale cross-sectional study
Source: Front Public Health. 2024 Mar 26;12:1357052. doi: 10.3389/fpubh.2024.1357052 (PMC11002127; doi:10.3389/fpubh.2024.1357052)
Supplement: Supplementary file 1 [file Data_Sheet_1.docx]

Supplementary Material

**Single and combined association between brominated flame retardants and cardiovascular disease: A large-scale cross-sectional study**

**Wenhao Yin^1^, Rui Xu^1^, Jiyu Zou^1^, Yaqin Wang^1^, Yan Zhang^2,*^**

^1^ Liaoning University of Traditional Chinese Medicine, Shenyang 110847, Liaoning, China

^2^ The Affiliated Hospital of Liaoning University of Traditional Chinese Medicine, Shenyang 110032, Liaoning, China

*** Correspondence:**Yan Zhang
Email address: yanzhang1016@126.com

**Keywords: brominated flame retardants, cardiovascular diseases, joint exposure, NHANES, cross-sectional study, PBB153.**

# Supplementary Figures and Tables

## Supplementary Tables

**Table S1.** Biological samples concentrations of BFRs in the study population.

| BFRs | MLOD  Serum (pg/g) | MLOD  Lipid (ng/g) | Detection  Ratio | All participants | Con | Case | P-value |
| --- | --- | --- | --- | --- | --- | --- | --- |
| 2005-2006 |  |  |  |  |  |  |  |
| PBB153 | 1.8 | 0.4 | 87% | 21.20 [11.20, 45.10] | 19.70 [10.90, 41.80] | 39.60 [22.20, 73.33] | <0.001 |
| PBDE28 | 1.8 | 0.4 | 99% | 11.00 [7.50, 15.20] | 11.00 [7.40, 14.90] | 13.40 [9.00, 19.60] | 0.008 |
| PBDE47 | 1.8 | 0.4 | 100% | 206.60 [136.10, 304.70] | 203.20 [136.10, 304.70] | 227.40 [147.62, 330.30] | 0.155 |
| PBDE85 | 1.8 | 0.4 | 83% | 4.20 [2.60, 6.60] | 4.10 [2.60, 6.60] | 4.30 [3.00, 6.70] | 0.491 |
| PBDE99 | 1.8 | 0.4 | 100% | 40.80 [27.10, 64.50] | 39.50 [27.00, 64.50] | 44.35 [29.20, 63.17] | 0.509 |
| PBDE100 | 1.8 | 0.4 | 100% | 40.30 [25.50, 57.40] | 39.70 [25.50, 56.70] | 45.50 [27.40, 66.60] | 0.246 |
| PBDE153 | 1.8 | 0.4 | 100% | 53.20 [34.80, 82.40] | 54.80 [34.90, 82.40] | 49.20 [31.52, 76.00] | 0.265 |
| PBDE154 | 1.8 | 0.4 | 81% | 3.90 [2.60, 6.40] | 3.90 [2.60, 6.40] | 4.25 [2.70, 6.30] | 0.218 |
| 2007-2008 |  |  |  |  |  |  |  |
| PBB153 | 1.8 | 0.4 | 92.7% | 23.00 [10.30, 40.20] | 22.00 [9.20, 39.40] | 32.30 [23.13, 42.45] | <0.001 |
| PBDE28 | 1.8 | 0.4 | 100% | 9.60 [6.60, 13.80] | 9.40 [6.50, 13.05] | 12.10 [8.60, 17.53] | <0.001 |
| PBDE47 | 1.8 | 0.4 | 100% | 155.70 [113.20, 256.40] | 150.20 [112.00, 247.30] | 208.00 [124.55, 297.80] | 0.001 |
| PBDE85 | 1.8 | 0.4 | 78% | 3.60 [1.80, 6.00] | 3.50 [1.80, 5.80] | 4.50 [2.95, 7.30] | 0.002 |
| PBDE99 | 1.8 | 0.4 | 100% | 31.70 [21.60, 55.50] | 31.40 [21.50, 54.35] | 40.40 [25.40, 70.80] | 0.003 |
| PBDE100 | 1.8 | 0.4 | 100% | 33.20 [22.00, 56.10] | 33.00 [21.60, 54.00] | 39.20 [22.98, 58.20] | 0.101 |
| PBDE153 | 1.8 | 0.4 | 100% | 54.90 [33.90, 101.30] | 54.90 [34.60, 101.50] | 52.15 [31.10, 89.88] | 0.119 |
| PBDE154 | 1.8 | 0.4 | 73% | 3.20 [1.80, 5.40] | 3.20 [1.80, 5.30] | 4.20 [2.52, 5.57] | 0.022 |
| 2009-2010 |  |  |  |  |  |  |  |
| PBB153 | 3.8 | 0.92 | 87% | 17.19 [7.86, 29.84] | 15.94 [7.06, 29.39] | 23.87 [18.69, 38.52] | <0.001 |
| PBDE28 | 2.8 | 0.66 | 98% | 7.44 [5.45, 11.33] | 7.43 [5.39, 11.10] | 9.32 [6.52, 14.16] | 0.001 |
| PBDE47 | 2.8 | 0.64 | 100% | 142.20 [93.40, 209.60] | 140.50 [93.24, 208.80] | 172.00 [98.09, 242.30] | 0.023 |
| PBDE85 | 1.9 | 0.44 | 89% | 2.66 [1.71, 3.94] | 2.63 [1.70, 3.93] | 2.89 [1.89, 4.99] | 0.042 |
| PBDE99 | 2.4 | 0.56 | 100% | 26.76 [17.71, 41.72] | 26.29 [17.65, 41.25] | 33.52 [19.15, 46.56] | 0.031 |
| PBDE100 | 1.9 | 0.44 | 100% | 29.16 [18.80, 40.97] | 28.82 [18.80, 40.26] | 30.97 [19.20, 46.84] | 0.138 |
| PBDE153 | 2.5 | 0.6 | 100% | 53.47 [36.00, 90.11] | 53.47 [36.53, 90.11] | 52.62 [34.77, 78.69] | 0.455 |
| PBDE154 | 2.5 | 0.58 | 80% | 2.51 [1.67, 3.84] | 2.51 [1.66, 3.74] | 2.74 [2.02, 4.34] | 0.015 |
| 2011-2012 |  |  |  |  |  |  |  |
| PBB153 | 0.7 | 0.18 | 94% | 12.02 [5.53, 23.05] | 11.23 [5.32, 22.29] | 26.33 [15.27, 47.81] | <0.001 |
| PBDE28 | 1.8 | 0.48 | 96% | 6.18 [4.68, 8.69] | 6.01 [4.64, 8.43] | 7.16 [5.44, 10.62] | 0.006 |
| PBDE47 | 2.7 | 0.70 | 100% | 111.20 [79.84, 179.10] | 109.30 [79.61, 173.10] | 136.60 [92.02, 205.30] | 0.006 |
| PBDE85 | 0.8 | 0.19 | 89% | 1.95 [1.37, 3.44] | 1.93 [1.37, 3.39] | 2.51 [1.50, 4.88] | 0.006 |
| PBDE99 | 1.4 | 0.37 | 100% | 21.14 [13.96, 35.53] | 20.85 [13.94, 33.23] | 30.66 [15.40, 50.28] | 0.003 |
| PBDE100 | 2.8 | 0.71 | 100% | 22.46 [15.30, 34.53] | 22.06 [15.26, 32.86] | 25.48 [16.83, 45.23] | 0.006 |
| PBDE153 | 0.7 | 0.18 | 100% | 46.87 [30.64, 82.69] | 46.71 [30.64, 80.96] | 61.69 [35.68, 94.40] | 0.067 |
| PBDE154 | 0.7 | 0.18 | 90% | 1.90 [1.35, 3.41] | 1.89 [1.34, 3.32] | 2.44 [1.47, 4.60] | 0.006 |
| 2013-2014 |  |  |  |  |  |  |  |
| PBB153 | 0.71 | 0.18 | 93% | 12.94 [5.13, 23.09] | 12.33 [4.85, 21.49] | 18.98 [14.34, 33.01] | <0.001 |
| PBDE28 | 0.71 | 0.18 | 100% | 6.49 [4.10, 9.25] | 6.08 [4.00, 9.14] | 7.86 [5.55, 12.71] | <0.001 |
| PBDE47 | 2.8 | 0.71 | 100% | 98.92 [69.91, 151.02] | 97.85 [68.70, 148.95] | 134.90 [94.59, 175.30] | <0.001 |
| PBDE85 | 0.71 | 0.18 | 88% | 2.00 [1.41, 3.36] | 1.99 [1.35, 3.26] | 2.62 [1.69, 4.22] | <0.001 |
| PBDE99 | 1.2 | 0.31 | 100% | 18.69 [12.93, 29.90] | 17.76 [12.75, 29.23] | 25.70 [15.11, 36.98] | <0.001 |
| PBDE100 | 0.71 | 0.18 | 100% | 21.16 [14.73, 31.18] | 20.64 [14.62, 30.59] | 25.56 [18.15, 39.06] | <0.001 |
| PBDE153 | 0.71 | 0.18 | 100% | 52.09 [33.77, 78.19] | 53.25 [32.85, 79.25] | 45.27 [36.69, 73.14] | 0.951 |
| PBDE154 | 0.71 | 0.18 | 88% | 1.97 [1.22, 2.80] | 1.91 [1.20, 2.78] | 2.35 [1.47, 3.53] | <0.001 |
| 2015-2016 |  |  |  |  |  |  |  |
| PBB153 | 1.1 | 0.28 | 83% | 10.81 [4.86, 19.46] | 10.06 [4.54, 18.40] | 18.40 [11.63, 23.12] | <0.001 |
| PBDE28 | 1.5 | 0.37 | 88% | 4.50 [2.92, 6.31] | 4.26 [2.92, 5.97] | 6.05 [4.66, 8.09] | <0.001 |
| PBDE47 | 1.3 | 0.31 | 100% | 80.88 [53.92, 107.70] | 79.37 [51.52, 105.90] | 99.01 [73.00, 159.80] | <0.001 |
| PBDE85 | 1.3 | 0.35 | 37% | 1.34 [1.34, 2.55] | 1.34 [1.34, 2.52] | 2.07 [1.34, 3.27] | <0.001 |
| PBDE99 | 1.1 | 0.30 | 100% | 15.24 [10.10, 23.37] | 15.10 [9.72, 22.03] | 19.72 [14.05, 33.05] | <0.001 |
| PBDE100 | 1.0 | 0.25 | 100% | 17.25 [11.48, 24.23] | 16.30 [11.18, 23.77] | 20.90 [14.19, 36.38] | <0.001 |
| PBDE153 | 1.0 | 0.25 | 100% | 52.26 [34.56, 82.16] | 50.34 [34.10, 77.73] | 66.48 [39.75, 114.60] | <0.001 |
| PBDE154 | 0.9 | 0.24 | 55% | 1.49 [0.92, 2.13] | 1.39 [0.92, 2.08] | 1.73 [0.92, 2.79] | <0.001 |

**Table S2**. The detection rate of the brominated flame retardants in NHANES 2005-2016.

| Exposures | The number of participants with BFRs at or above LOD | The number of participants with BFRs below LOD | Detection rate (%) |
| --- | --- | --- | --- |
| PBDE17 | 799 | 11574 | 6.46 |
| PBDE28 | 12035 | 378 | 96.95 |
| PBDE47 | 12421 | 0 | 100.00 |
| PBDE66 | 1734 | 10687 | 13.96 |
| PBDE85 | 9655 | 2766 | 77.73 |
| PBDE99 | 12421 | 0 | 100.00 |
| PBDE100 | 12421 | 0 | 100.00 |
| PBDE153 | 12421 | 0 | 100.00 |
| PBDE154 | 9687 | 2718 | 78.09 |
| PBDE183 | 5315 | 7106 | 42.79 |
| PBDE209 | 8598 | 3799 | 69.36 |
| PBB153 | 11115 | 1298 | 89.54 |

**Table S3**. Weighted associations of with ln-transformed BFRs in lipid adjusted with CVD in all participants

|  | Cardiovascular disease, OR (95%CI) | | |
| --- | --- | --- | --- |
|  | Model1 ^a^ | Model2 ^b^ | Model3 ^c^ |
| PBB153 |  |  |  |
| Q1 | Ref (1.0) | Ref (1.0) | Ref (1.0) |
| Q2 | 7.96 (4.40, 14.0) ^***^ | 3.87 (1.99, 7.53) ^***^ | 3.73 (1.94, 7.20) ^***^ |
| Q3 | 16.8 (9.31, 30.3) ^***^ | 5.56 (2.85, 10.9) ^***^ | 4.72 (2.47, 9.04) ^***^ |
| Q4 | 19.0 (10.4, 34.7) ^***^ | 5.60 (2.75, 11.4) ^***^ | 4.55 (2.26, 9.16) ^***^ |
| PBDE28 |  |  |  |
| Q1 | Ref (1.0) | Ref (1.0) | Ref (1.0) |
| Q2 | 1.86 (1.28, 2.71) ^**^ | 1.42 (0.96, 2.12) | 1.37 (0.87, 2.17) |
| Q3 | 1.86 (1.24, 2.79) ^**^ | 1.40 (0.90, 2.18) | 1.30 (0.82, 2.08) |
| Q4 | 3.13 (2.15, 4.56) ^***^ | 1.88 (1.26, 2.82) ^**^ | 1.74 (1.12, 2.70) ^*^ |
| PBDE47 |  |  |  |
| Q1 | Ref (1.0) | Ref (1.0) | Ref (1.0) |
| Q2 | 1.14 (0.82, 1.59) | 1.04 (0.73, 1.49) | 1.01 (0.69, 1.46) |
| Q3 | 1.41 (0.97, 2.03) | 1.27 (0.86, 1.87) | 1.14 (0.75, 1.74) |
| Q4 | 2.20 (1.56, 3.11) ^***^ | 1.53 (1.05, 2.22) ^*^ | 1.42 (0.94, 2.14) |
| PBDE85 |  |  |  |
| Q1 | Ref (1.0) | Ref (1.0) | Ref (1.0) |
| Q2 | 0.84 (0.59, 1.20) | 0.96 (0.66, 1.38) | 0.98 (0.67, 1.43) |
| Q3 | 1.37 (0.99, 1.91) | 1.23 (0.86, 1.76) | 1.18 (0.80, 1.75) |
| Q4 | 1.78 (1.26, 2.52) ^**^ | 1.45 (1.03, 2.04) ^*^ | 1.32 (0.91, 1.92) |
| PBDE99 |  |  |  |
| Q1 | Ref (1.0) | Ref (1.0) | Ref (1.0) |
| Q2 | 0.71 (0.48, 1.06) | 0.75 (0.50, 1.11) | 0.75 (0.49, 1.16) |
| Q3 | 1.49 (1.06, 2.09) ^*^ | 1.37 (0.95, 1.98) | 1.33 (0.90, 1.97) |
| Q4 | 1.62 (1.16, 2.26) ^**^ | 1.27 (0.90, 1.81) | 1.20 (0.82, 1.77) |
| PBDE100 |  |  |  |
| Q1 | Ref (1.0) | Ref (1.0) | Ref (1.0) |
| Q2 | 1.21 (0.88, 1.65) | 1.16 (0.83, 1.61) | 1.09 (0.75, 1.59) |
| Q3 | 1.27 (0.88, 1.83) | 1.20 (0.81, 1.77) | 1.12 (0.74, 1.69) |
| Q4 | 1.74 (1.25, 2.41) ^**^ | 1.39 (0.98, 1.97) | 1.21 (0.81, 1.81) |
| PBDE153 |  |  |  |
| Q1 | Ref (1.0) | Ref (1.0) | Ref (1.0) |
| Q2 | 0.75 (0.53, 1.06) | 0.85 (0.58, 1.25) | 0.77 (0.52, 1.51) |
| Q3 | 0.63 (0.48, 0.84) ^**^ | 0.78 (0.57, 1.05) | 0.76 (0.55, 1.05) |
| Q4 | 0.75 (0.54, 1.05) | 0.76 (0.54, 1.06) | 0.69 (0.48, 1.00) ^*^ |
| PBDE154 |  |  |  |
| Q1 | Ref (1.0) | Ref (1.0) | Ref (1.0) |
| Q2 | 1.15 (0.84, 1.58) | 0.99 (0.71, 1.38) | 1.00 (0.71, 1.39) |
| Q3 | 1.41 (0.97, 2.05) | 1.10 (0.74, 1.64) | 1.02 (0.67, 1.58) |
| Q4 | 1.85 (1.31, 2.62) | 1.39 (0.98, 1.97) | 1.29 (0.87, 1.91) |

^a^ Model 1: crude model

^b^ Model 2: adjusted for age, gender, race, and education

^c^ Model 3: Model 2 + physical activity + ln-transformed cotinine + alcohol consumption + family history of CVD + diabetes + hypertension disorders + energy intake + BMI + PIR

^***^ *p*<0.001; ^**^ *p*<0.01; ^*^ *p*<0.05

**Table S4**. Weighted associations of ln-transformed serum BFRs/ lipid-adjusted BFRs with CVD in all participants stratified by CVD subtype.

|  | Cardiovascular disease, OR (95%CI) | | | | |
| --- | --- | --- | --- | --- | --- |
|  | congestive heart failure | coronary heart disease | angina pectoris | heart attack | stroke |
| Ln-transformed serum BFRs | |  |  |  |  |
| PBB153 | 1.23 (1.02, 1.49) ^*^ | 1.29 (1.06, 1.56) ^*^ | 1.19 (0.96, 1.48) | 1.17 (0.98, 1.40) | 1.22 (0.95, 1.56) |
| PBDE28 | 1.19 (0.90, 1.58) | 1.26 (0.84, 1.88) | 1.13 (0.80, 1.60) | 1.10 (0.84, 1.44) | 1.29 (0.89, 1.87) |
| PBDE47 | 1.25 (0.98, 1.59) | 1.08 (0.80, 1.46) | 0.99 (0.74, 1.33) | 1.13 (0.80, 1.34) | 1.27 (0.96, 1.69) |
| PBDE85 | 1.23 (1.00, 1.52) | 1.03 (0.79, 1.36) | 0.92 (0.69, 1.21) | 0.99 (0.78, 1.27) | 1.16 (0.91, 1.47) |
| PBDE99 | 1.29 (1.06, 1.58) ^*^ | 1.04 (0.82, 1.32) | 0.96 (0.76, 1.22) | 1.03 (0.82, 1.30) | 1.22 (0.98, 1.51) |
| PBDE100 | 1.21 (0.93, 1.58) | 1.02 (0.75, 1.39) | 0.91 (0.67, 1.26) | 1.03 (0.81, 1.33) | 1.17 (0.89, 1.55) |
| PBDE153 | 1.00 (0.71, 1.41) | 0.89 (0.63, 1.25) | 0.84 (0.56, 1.25) | 0.97 (0.74, 1.28) | 1.12 (0.80, 1.56) |
| PBDE154 | 1.29 (1.02, 1.63) ^*^ | 1.02 (0.78, 1.33) | 0.91 (0.69, 1.22) | 1.01 (0.79, 1.29) | 1.15 (0.90, 1.46) |
| Ln-transformed lipid-adjusted BFRs | |  |  |  |  |
| PBB153 | 1.25 (1.03, 1.52) ^*^ | 1.31 (1.09, 1.57) ^**^ | 1.20 (0.95, 1.50) | 1.20 (1.00, 1.44) | 1.24 (0.96, 1.59) |
| PBDE28 | 1.22 (0.91, 1.64) | 1.29 (0.84, 1.98) | 1.13 (0.78, 1.65) | 1.14 (0.85, 1.52) | 1.34 (0.92, 1.94) |
| PBDE47 | 1.28 (1.00, 1.64) | 1.09 (0.80, 1.49) | 0.98 (0.72, 1.33) | 1.06 (0.81, 1.38) | 1.30 (0.98, 1.72) |
| PBDE85 | 1.26 (1.01, 1.56) ^*^ | 1.03 (0.78, 1.37) | 0.90 (0.67, 1.21) | 1.00 (0.78, 1.29) | 1.17 (0.93, 1.48) |
| PBDE99 | 1.32 (1.08, 1.61) ^**^ | 1.05 (0.82, 1.34) | 0.95 (0.74, 1.22) | 1.05 (0.83, 1.33) | 1.23 (1.00, 1.53) |
| PBDE100 | 1.23 (0.94, 1.63) | 1.03 (0.75, 1.41) | 0.90 (0.64, 1.27) | 1.06 (0.81, 1.37) | 1.19 (0.92, 1.55) |
| PBDE153 | 1.01 (0.70, 1.46) | 0.89 (0.63, 1.25) | 0.82 (0.54, 1.24) | 0.99 (0.74, 1.31) | 1.13 (0.82, 1.56) |
| PBDE154 | 1.34 (1.05, 1.70) ^*^ | 1.02 (0.77, 1.35) | 0.91 (0.67, 1.23) | 1.03 (0.80, 1.32) | 1.17 (0.93, 1.48) |

Note: CVD: Cardiovascular disease; PIR: Ratio of family income to poverty; BMI: Body mass index;

Model: adjusted for age, gender, race, education, physical activity, ln-transformed cotinine, alcohol consumption, family history of CVD, diabetes, hypertension disorders, energy intake, BMI, and PIR.

^***^ *p*<0.001; ^**^ *p*<0.01; ^*^ *p*<0.

**Table S5**. Weighted association between ln-transformed concentration of PBB153 and CVD stratified by covariates.

| Covariates | Ln-transformed concentration of PBB153 | | | | |
| --- | --- | --- | --- | --- | --- |
|  | Q1 | Q2 | Q3 | Q4 | *P*_int_ |
| Age |  |  |  |  | >0.9 |
| <50 | Ref (1.0) | 3.74 (1.54, 9.07) ^**^ | 5.74 (2.31, 14.3) ^***^ | 4.58 (1.78, 11.8) ^**^ |  |
| ≥50 | Ref (1.0) | 3.15 (1.06, 9.32) ^*^ | 4.13 (1.35, 12.6) ^*^ | 3.93 (1.32, 11.7) ^*^ |  |
| Gender |  |  |  |  | 0.11 |
| Male | Ref (1.0) | 3.70 (1.54, 8.89) ^**^ | 5.49 (2.23, 13.5) ^***^ | 4.11 (1.72, 9.78) ^**^ |  |
| Female | Ref (1.0) | 2.88 (1.01, 8.22) ^*^ | 3.92 (1.33, 11.5) ^*^ | 4.15 (1.45, 11.9) ^**^ |  |
| BMI |  |  |  |  | 0.03 ^*^ |
| <24.9 | Ref (1.0) | 1.21 (0.34, 4.32) | 3.75 (1.14, 12.4) ^*^ | 3.80 (1.02, 14.1) ^*^ |  |
| 24.9-<29.9 | Ref (1.0) | 4.68 (1.02, 21.4) ^*^ | 8.34 (1.75, 39.8) ^**^ | 6.79 (1.43, 32.2) ^*^ |  |
| ≥29.9 | Ref (1.0) | 4.97 (1.90, 13.0) ^**^ | 4.63 (1.71, 12.5) ^**^ | 4.87 (1.74, 13.6) ^**^ |  |
| Physical activity |  |  |  |  | 0.6 |
| No | Ref (1.0) | 3.49 (1.07, 1.4) ^*^ | 3.57 (1.15, 11.1) ^*^ | 5.43 (1.73, 17.1) ^**^ |  |
| Lower intensity | Ref (1.0) | 4.93 (1.72, 14.1) ^**^ | 8.00 (3.21, 19.9) ^***^ | 5.87 (2.36, 14.6) ^***^ |  |
| Higher intensity | Ref (1.0) | 2.06 (0.45, 9.34) | 2.76 (0.61, 12.6) | 2.98 (0.64, 13.9) |  |
| Energy intake, |  |  |  |  | 0.06 |
| Low | Ref (1.0) | 3.28 (1.05, 10.3) ^*^ | 3.45 (1.15, 10.4) ^*^ | 5.24 (1.74, 15.8) ^**^ |  |
| Adequate | Ref (1.0) | 4.87 (1.70, 13.9) ^**^ | 7.75 (3.16, 19.0) ^***^ | 5.60 (2.27, 13.8) ^***^ |  |
| High | Ref (1.0) | 1.74 (0.38, 7.89) | 2.64 (0.57, 12.3) | 2.45 (0.53, 11.2) |  |
| Alcohol consumption |  |  |  |  | >0.9 |
| Yes | Ref (1.0) | 4.93 (1.71, 14.2) ^**^ | 8.09 (3.21, 20.4) ^***^ | 6.15 (2.46, 15.4) ^***^ |  |
| No | Ref (1.0) | 3.43 (1.09, 10.8) ^*^ | 3.69 (1.23, 11.1) ^*^ | 6.21 (2.03, 19.0) ^**^ |  |
| Family history of CVD |  |  |  |  | 0.2 |
| Yes | Ref (1.0) | 8.66 (2.60, 28.8) ^***^ | 7.83 (2.20, 27.8) ^**^ | 9.22 (2.63, 32.4) ^***^ |  |
| No | Ref (1.0) | 2.50 (1.17, 5.34) ^*^ | 4.13 (1.83, 9.34) ^***^ | 4.08 (1.80, 9.21) ^***^ |  |
| Diabetes |  |  |  |  | <0.001 ^***^ |
| Yes | Ref (1.0) | 1.45 (0.45, 4.64) | 1.37 (0.44, 4.25) | 1.19 (0.38, 3.73) |  |
| No | Ref (1.0) | 4.64 (2.10, 10.3) ^***^ | 7.91 (3.71, 16.8) ^***^ | 8.71 (4.00, 19.0) ^***^ |  |
| Hypertension disorders |  |  |  |  | 0.5 |
| Yes | Ref (1.0) | 3.56 (1.62, 7.84) ^**^ | 5.21 (2.54, 10.7) ^***^ | 5.06 (2.47, 10.4) ^***^ |  |
| No | Ref (1.0) | 3.69 (1.19, 11.4) ^*^ | 4.48 (1.45, 13.8) ^*^ | 5.26 (1.64, 16.9) ^**^ |  |

Note: CVD: Cardiovascular disease; PIR: Ratio of family income to poverty; BMI: Body mass index;

Model: adjusted for age, gender, race, education, physical activity, ln-transformed cotinine, alcohol consumption, family history of CVD, diabetes, hypertension disorders, energy intake, BMI, and PIR.

^***^ *p*<0.001; ^**^ *p*<0.01; ^*^ *p*<0.05

Table S6. Weighted association between ln-transformed concentration of PBB153 in lipid and CVD stratified by covariates.

| Covariates | ln-transformed concentration of PBB153 in lipid | | | | |
| --- | --- | --- | --- | --- | --- |
|  | Q1 | Q2 | Q3 | Q4 | *P*_int_ |
| Age |  |  |  |  | >0.9 |
| <50 | Ref (1.0) | 3.85 (1.53, 9.66) ^**^ | 5.61 (2.25, 14.0) ^***^ | 4.23 (1.58, 11.3) ^**^ |  |
| ≥50 | Ref (1.0) | 2.70 (0.96, 7.57) | 3.19 (1.10, 9.26) ^*^ | 3.13 (1.10, 8.88) ^*^ |  |
| Gender |  |  |  |  | 0.15 |
| Male | Ref (1.0) | 6.16 (2.51, 15.1) ^***^ | 9.38 (2.85, 30.9) ^***^ | 9.15 (2.73, 30.6) ^***^ |  |
| Female | Ref (1.0) | 3.21 (1.34, 7.70) ^*^ | 3.82 (1.58, 9.23) ^**^ | 3.28 (1.34, 8.04) ^*^ |  |
| BMI |  |  |  |  | 0.005 ^**^ |
| <24.9 | Ref (1.0) | 0.70 (0.18, 2.72) | 4.13 (1.34, 12.7) ^*^ | 3.61 (1.03, 12.7) ^*^ |  |
| 24.9-<29.9 | Ref (1.0) | 5.20 (1.09, 24.8) ^*^ | 9.06 (1.97, 41.7) ^**^ | 7.04 (1.45, 34.1) ^*^ |  |
| ≥29.9 | Ref (1.0) | 5.14 (1.81, 14.6) ^**^ | 3.61 (1.26, 10.3) ^*^ | 4.17 (1.38, 12.6) ^*^ |  |
| Physical activity |  |  |  |  | 0.2 |
| No | Ref (1.0) | 3.91 (1.22, 12.5) ^*^ | 3.68 (1.13, 12.0) ^*^ | 6.57 (2.05, 21.1) ^**^ |  |
| Lower intensity | Ref (1.0) | 4.09 (1.54, 10.9) ^**^ | 6.74 (2.94, 15.5) ^***^ | 4.87 (2.10, 11.3) ^***^ |  |
| Higher intensity | Ref (1.0) | 2.66 (0.60, 11.8) | 2.85 (0.62, 13.0) | 2.37 (0.50, 11.2) |  |
| Energy intake, |  |  |  |  | 0.076 |
| Low | Ref (1.0) | 3.11 (0.98, 9.86) | 5.21 (1.83, 14.8) ^**^ | 5.46 (1.98, 15.0) ^**^ |  |
| Adequate | Ref (1.0) | 3.99 (1.56, 10.2) ^**^ | 3.49 (1.20, 10.1) ^*^ | 3.28 (1.13, 9.52) ^*^ |  |
| High | Ref (1.0) | 5.35 (0.95, 30.1) | 7.07 (1.33, 37.7) ^*^ | 5.16 (0.68, 39.1) |  |
| Alcohol consumption |  |  |  |  | >0.9 |
| Yes | Ref (1.0) | 5.43 (2.40, 12.3) ^***^ | 7.72 (3.36, 17.7) ^***^ | 7.14 (3.09, 16.5) ^***^ |  |
| No | Ref (1.0) | 1.92 (0.62, 5.91) | 2.27 (0.78, 6.58) | 2.86 (0.97, 8.47) |  |
| Family history of CVD |  |  |  |  | 0.043 ^*^ |
| Yes | Ref (1.0) | 7.73 (2.49, 24.0) ^***^ | 7.46 (2.43, 22.9) ^***^ | 6.64 (2.10, 21.0) ^**^ |  |
| No | Ref (1.0) | 2.49 (1.18, 5.26) ^*^ | 3.96 (1.77, 8.86) ^**^ | 4.12 (1.83, 9.30) ^***^ |  |
| Diabetes |  |  |  |  | <0.001 ^***^ |
| Yes | Ref (1.0) | 1.60 (0.45, 5.77) | 1.06 (0.30, 3.71) | 1.17 (033, 4.10) |  |
| No | Ref (1.0) | 4.43 (2.02, 9.72) ^***^ | 8.24 (3.90, 17.4) ^***^ | 8.09 (3.77, 17.4) ^***^ |  |
| Hypertension disorders |  |  |  |  | 0.2 |
| Yes | Ref (1.0) | 3.19 (1.50, 6.81) ^**^ | 4.56 (2.31, 9.02) ^***^ | 4.25 (2.14, 8.43) ^***^ |  |
| No | Ref (1.0) | 3.90 (1.26, 12.1) ^*^ | 4.54 (1.52, 13.5) ^**^ | 5.58 (1.72, 18.1) ^**^ |  |

Note: CVD: Cardiovascular disease; PIR: Ratio of family income to poverty; BMI: Body mass index;

Model: adjusted for age, gender, race, education, physical activity, ln-transformed cotinine, alcohol consumption, family history of CVD, diabetes, hypertension disorders, energy intake, BMI, and PIR.

^***^ *p*<0.001; ^**^ *p*<0.01; ^*^ *p*<0.05

**Table S7.** Weighted association between ln-transformed concentration of PBDE28 in lipid and CVD stratified by covariates.

| Covariates | ln-transformed concentration of PBDE28 in lipid | | | | |
| --- | --- | --- | --- | --- | --- |
|  | Q1 | Q2 | Q3 | Q4 | *P*_int_ |
| Age |  |  |  |  | >0.9 |
| <50 | Ref (1.0) | 2.10 (1.08, 4.09) ^*^ | 1.29 (0.55, 3.03) | 1.78 (0.77, 4.08) |  |
| ≥50 | Ref (1.0) | 1.16 (0.67, 1.99) | 1.22 (0.71, 2.09) | 1.58 (0.95, 2.62) |  |
| Gender |  |  |  |  | 0.9 |
| Male | Ref (1.0) | 1.70 (0.89, 3.23) | 1.64 (0.86, 3.15) | 1.86 (0.97, 3.54) |  |
| Female | Ref (1.0) | 1.09 (0.64, 1.86) | 1.09 (0.61, 1.95) | 1.71 (0.98, 2.98) |  |
| BMI |  |  |  |  | 0.018^*^ |
| <24.9 | Ref (1.0) | 2.09 (0.79, 5.50) | 2.46 (0.94, 6.48) | 2.79 (1.21, 6.41) ^*^ |  |
| 24.9-<29.9 | Ref (1.0) | 1.64 (0.73, 3.70) | 1.35 (0.57, 3.20) | 1.97 (0.90, 4.27) |  |
| ≥29.9 | Ref (1.0) | 1.12 (0.59, 2.13) | 1.05 (0.58, 1.91) | 1.31 (0.73, 2.34) |  |
| Physical activity |  |  |  |  | 0.2 |
| No | Ref (1.0) | 1.21 (0.56, 2.61) | 2.19 (1.02, 4.69) ^*^ | 2.01 (1.00, 4.04) ^*^ |  |
| Lower intensity | Ref (1.0) | 1.52 (0.74, 3.11) | 1.23 (0.62, 2.47) | 2.05 (1.12, 3.78) ^*^ |  |
| Higher intensity | Ref (1.0) | 1.21 (0.43, 3.40) | 0.51 (0.16, 1.59) | 0.84 (0.29, 2.42) |  |
| Energy intake, |  |  |  |  | >0.9 |
| Low | Ref (1.0) | 1.69 (0.95, 3.01) | 1.15 (0.69, 1.93) | 2.04 (1.21, 3.43) ^**^ |  |
| Adequate | Ref (1.0) | 0.94 (0.45, 1.97) | 1.37 (0.63, 2.94) | 1.29 (0.58, 2.86) |  |
| High | Ref (1.0) | 3.45 (0.71, 16.9) | 2.11 (0.47, 9.45) | 3.80 (0.72, 20.0) |  |
| Alcohol consumption |  |  |  |  | 0.6 |
| Yes | Ref (1.0) | 1.55 (0.90, 2.66) | 1.56 (0.87, 2.79) | 1.88 (1.07, 3.28) ^*^ |  |
| No | Ref (1.0) | 1.11 (0.53, 2.31) | 0.88 (0.45, 1.72) | 1.63 (0.84, 3.14) |  |
| Family history of CVD |  |  |  |  | 0.047^*^ |
| Yes | Ref (1.0) | 1.39 (0.66, 2.93) | 1.35 (0.60, 3.06) | 1.08 (0.57, 2.05) |  |
| No | Ref (1.0) | 1.38 (0.82, 2.32) | 1.31 (0.77, 2.23) | 2.01 (1.17, 3.44) ^*^ |  |
| Diabetes |  |  |  |  | 0.8 |
| Yes | Ref (1.0) | 1.16 (0.44, 3.06) | 1.68 (0.67, 4.17) | 1.72 (0.75, 3.95) |  |
| No | Ref (1.0) | 1.45 (0.93, 2.26) | 1.13 (0.68, 1.87) | 1.76 (1.07, 2.89) ^*^ |  |
| Hypertension disorders |  |  |  |  | 0.3 |
| Yes | Ref (1.0) | 1.38 (0.79, 2.40) | 1.23 (0.73, 2.10) | 1.60 (0.94, 2.72) |  |
| No | Ref (1.0) | 1.59 (0.72, 3.53) | 1.45 (0.66, 3.21) | 2.34 (1.14, 4.81) ^*^ |  |

Note: CVD: Cardiovascular disease; PIR: Ratio of family income to poverty; BMI: Body mass index;

Model: adjusted for age, gender, race, education, physical activity, ln-transformed cotinine, alcohol consumption, family history of CVD, diabetes, hypertension disorders, energy intake, BMI, and PIR.

^***^ *p*<0.001; ^**^ *p*<0.01; ^*^ *p*<0.05

**Table S8**. Weighted association between ln-transformed concentration of PBDE153 in lipid and CVD stratified by covariates.

|  | ln-transformed concentration of PBDE153 in lipid | | | | |
| --- | --- | --- | --- | --- | --- |
|  | Q1 | Q2 | Q3 | Q4 | *P*_int_ |
| Age |  |  |  |  | 0.4 |
| <50 | Ref (1.0) | 0.87 (0.38, 1.99) | 1.04 (0.46, 2.38) | 0.61 (0.26, 1.45) |  |
| ≥50 | Ref (1.0) | 1.17 (0.75, 1.81) | 1.08 (0.70, 1.66) | 1.33 (0.87, 2.04) |  |
| Gender |  |  |  |  | 0.4 |
| Male | Ref (1.0) | 0.90 (0.54, 1.51) | 0.91 (0.49, 1.68) | 1.02 (0.60, 1.73) |  |
| Female | Ref (1.0) | 1.28 (0.73, 2.26) | 1.35 (0.80, 2.29) | 1.46 (0.82, 2.61) |  |
| BMI |  |  |  |  | 0.071 |
| <24.9 | Ref (1.0) | 0.99 (0.41, 2.37) | 1.17 (0.42, 3.29) | 1.88 (0.96, 3.67) |  |
| 24.9-<29.9 | Ref (1.0) | 0.82 (0.40, 1.70) | 0.89 (0.46, 1.74) | 1.10 (0.57, 2.16) |  |
| ≥29.9 | Ref (1.0) | 1.36 (0.77, 2.41) | 1.21 (0.67, 2.18) | 1.03 (0.55, 1.91) |  |
| Physical activity |  |  |  |  | 0.074 |
| No | Ref (1.0) | 1.82 (0.92, 3.60) | 2.11 (1.00, 4.44) ^*^ | 2.19 (1.12, 4.31) ^*^ |  |
| Lower intensity | Ref (1.0) | 0.76 (0.45, 1.27) | 0.96 (0.56, 1.64) | 1.12 (0.67, 1.88) |  |
| Higher intensity | Ref (1.0) | 1.51 (0.55, 4.17) | 0.59 (0.16, 2.22) | 0.75 (0.26, 2.19) |  |
| Energy intake, |  |  |  |  | 0.2 |
| Low | Ref (1.0) | 1.14 (0.62, 2.11) | 1.21 (0.73, 2.01) | 1.51 (0.87, 2.61) |  |
| Adequate | Ref (1.0) | 1.06 (0.60, 1.88) | 1.02 (0.52, 2.02) | 1.04 (0.54, 2.02) |  |
| High | Ref (1.0) | 0.82 (0.20, 3.29) | 1.03 (0.26, 4.08) | 0.79 (0.22, 2.81) |  |
| Alcohol consumption |  |  |  |  | 0.7 |
| Yes | Ref (1.0) | 1.19 (0.76, 1.85) | 1.15 (0.68, 1.94) | 1.25 (0.78, 2.00) |  |
| No | Ref (1.0) | 0.99 (0.54, 1.84) | 1.24 (0.66, 2.34) | 1.44 (0.79, 2.64) |  |
| Family history of CVD |  |  |  |  | 0.007 |
| Yes | Ref (1.0) | 0.89 (0.41, 1.92) | 0.88 (0.37, 2.11) | 0.62 (0.32, 1.21) |  |
| No | Ref (1.0) | 1.24 (0.81, 1.90) | 1.29 (0.82, 2.02) | 1.61 (1.03, 2.52) ^*^ |  |
| Diabetes |  |  |  |  | 0.7 |
| Yes | Ref (1.0) | 1.05 (0.49, 2.24) | 1.37 (0.68, 2.77) | 1.19 (0.57, 2.45) |  |
| No | Ref (1.0) | 1.09 (0.70, 1.69) | 1.00 (0.63, 1.58) | 1.32 (0.85, 2.06) |  |
| Hypertension disorders |  |  |  |  | 0.4 |
| Yes | Ref (1.0) | 1.19 (0.75, 1.89) | 1.16 (0.71, 1.89) | 1.19 (0.73, 1.94) |  |
| No | Ref (1.0) | 0.98 (0.57, 1.67) | 1.18 (0.57, 2.42) | 1.51 (0.84, 2.72) |  |

Note: CVD: Cardiovascular disease; PIR: Ratio of family income to poverty; BMI: Body mass index;

Model: adjusted for age, gender, race, education, physical activity, ln-transformed cotinine, alcohol consumption, family history of CVD, diabetes, hypertension disorders, energy intake, BMI, and PIR.

^***^ *p*<0.001; ^**^ *p*<0.01; ^*^ *p*<0.05

## Supplementary Figures

**Supplementary Figure 1.** the correlations across the various BFRs (A) ln-transformed concentrations of BFRs; (B) ln-transformed concentrations of BFRs in lipid.

**Supplementary Figure 2.** Non-linear association between BFRs and CVD. (A) ln-transformed concentrations of PBB153; (A) ln-transformed concentrations of PBDE28; (B) ln-transformed concentrations of PBDE47; (C) ln-transformed concentrations of PBDE85; (D) ln-transformed concentrations of PBDE99; (E) ln-transformed concentrations of PBDE100; (F) ln-transformed concentrations of PBDE100; (G) ln-transformed concentrations of PBDE153; (H) ln-transformed concentrations of PBDE154.

**Supplementary Figure 3.** Non-linear association between BFRs in lipid and CVD. (A) ln-transformed concentrations of PBB153; (A) ln-transformed concentrations of PBDE28; (B) ln-transformed concentrations of PBDE47; (C) ln-transformed concentrations of PBDE85; (D) ln-transformed concentrations of PBDE99; (E) ln-transformed concentrations of PBDE100; (F) ln-transformed concentrations of PBDE100; (G) ln-transformed concentrations of PBDE153; (H) ln-transformed concentrations of PBDE154.
