# Supplementary figures and images for: Single and combined association between brominated flame retardants and cardiovascular disease: a large-scale cross-sectional study
Source: Front Public Health. 2024 Mar 26;12:1357052. doi: 10.3389/fpubh.2024.1357052 (PMC11002127; doi:10.3389/fpubh.2024.1357052)

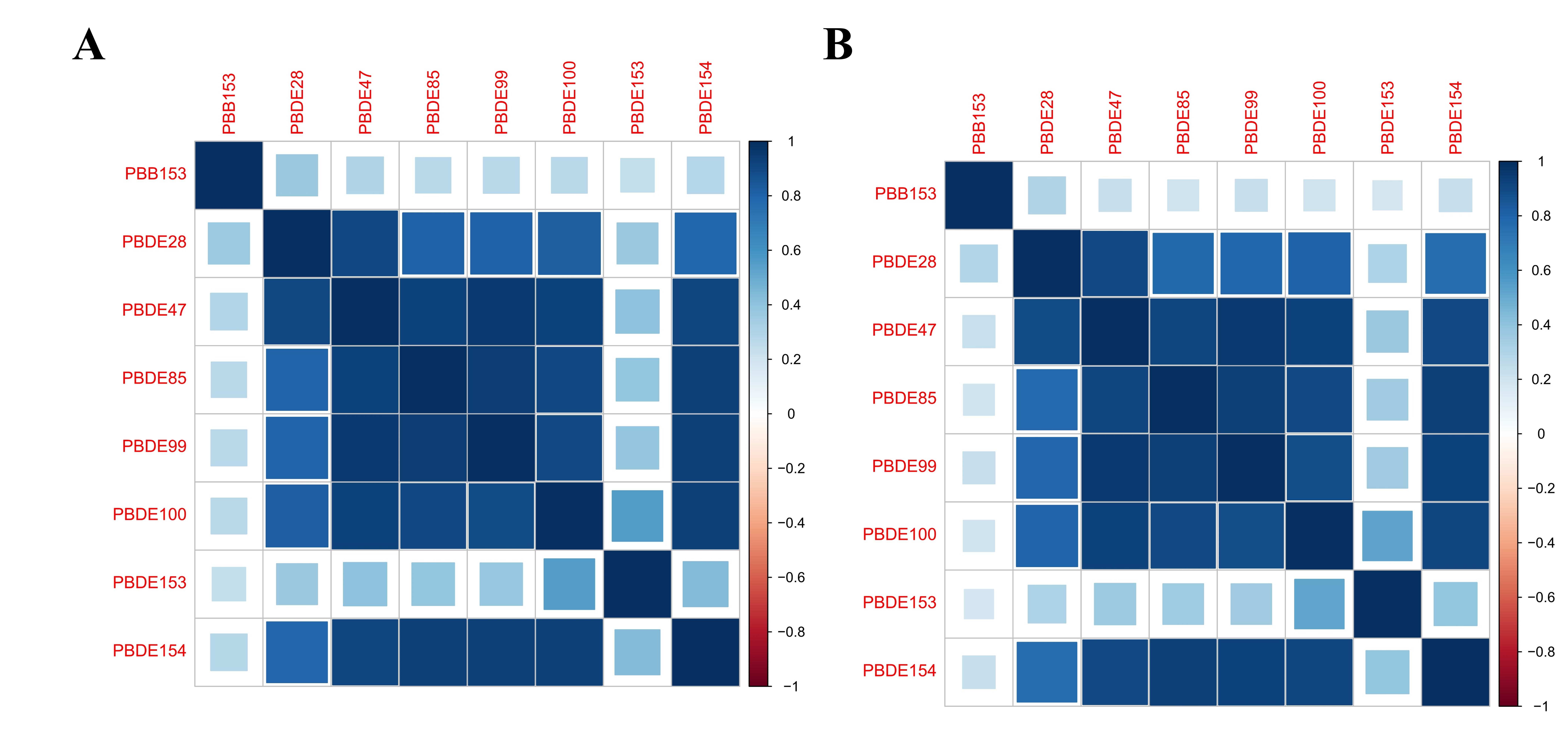

Supplement: Supplementary file 2 [file Presentation_1.ZIP › Supplement_Figure/Figure S1.tif]

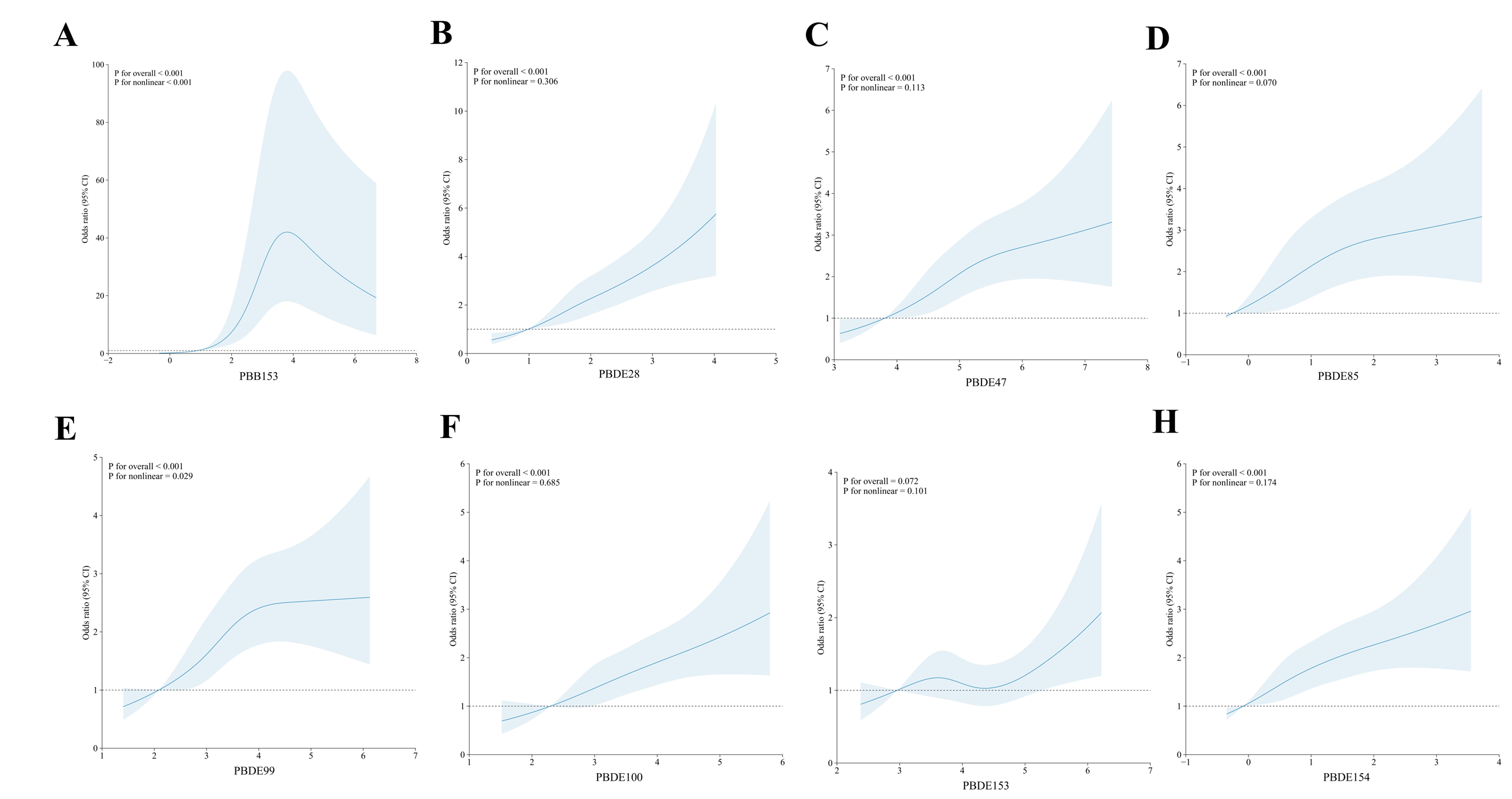

Supplement: Supplementary file 2 [file Presentation_1.ZIP › Supplement_Figure/Figure S2.tif]

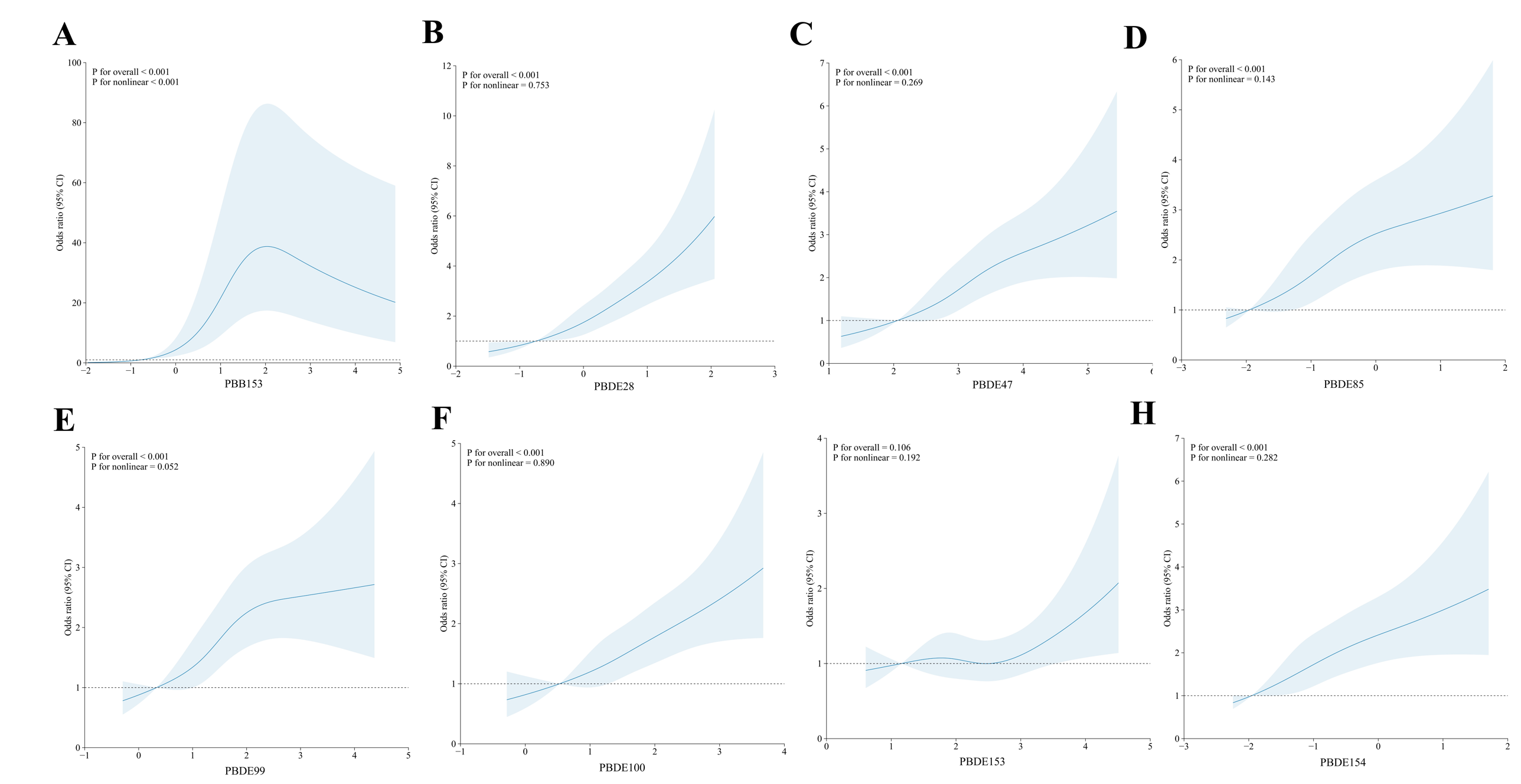

Supplement: Supplementary file 2 [file Presentation_1.ZIP › Supplement_Figure/Figure S3.tif]
